# Development of a proof of concept immunochromatographic lateral flow assay for point of care diagnosis of *Mycobacterium tuberculosis*

**DOI:** 10.1186/1756-0500-6-202

**Published:** 2013-05-21

**Authors:** Liya Wassie, Markos Abebe, Abraham Aseffa, Kidist Bobosha, Martha Zewdie, Menberwork Chanyalew, Lawrence K Yamuah, Arantxa Cortés, Jose R González, Jose M Delgado, Ismail Ceyhan, Ida Rosenkrands, Karin Weldingh, Peter Andersen, Timothy Mark Doherty

**Affiliations:** 1 Armauer Hansen Research Institute, P.O. Box: 1005, Jimma Road, Addis Ababa, Ethiopia; 2 Vircell, S.I., 18320 Santa Fe, Granada, Spain; 3 Refik Saydam National Institute of Hygiene, Cemal Gürsel Cad. No: 18, Sihhiye, Turkey; 4 Department of Infectious Disease Immunology, Statens Serum Institute, 5 Artillertivej, DK-2300, Copenhagen S, Denmark; 5 Current address: GlaxoSmithKline, Nykær 68, Copenhagen, Denmark

**Keywords:** IgG, Antibody, TB, Antigen, Serology, Diagnosis, ELISA

## Abstract

**Background:**

Despite major public health initiatives and the existence of efficacious treatment regimes, tuberculosis (TB) remains a threat, particularly in resource-limited settings. A significant part of the problem is the difficulty of rapidly identifying infected individuals, and as a result, there has been renewed interest in developing better diagnostics for infection or disease caused by *Mycobacterium tuberculosis*. Many of the existing tools, however, have limitations such as poor sensitivity or specificity, or the need for well-equipped laboratories to function effectively. Serodiagnostic approaches in particular have long drawn attention, due to their potential utility in large field studies, particularly in resource-poor settings. Unfortunately none of the serodiagnostic approaches have so far proven useful under field conditions.

**Results:**

We screened a large panel of antigens with serodiagnostic potential by ELISA and selected a subpanel that was strongly and broadly recognised by TB patients, but not by controls. These antigens were then formulated into a simple immuno-chromatographic lateral flow assay format, suitable for field use, and tested against panels of plasma and blood samples from individuals with different clinical status (confirmed TB patients, household contacts, and apparently healthy community controls), recruited from Ethiopia (a highly TB-endemic country) and Turkey (a TB meso-endemic country). While specificity was good (97-100% in non TB-endemic controls), the sensitivity was not as high as expected (46-54% in pulmonary TB, 25-29% in extra-pulmonary TB).

**Conclusions:**

Though below the level of sensitivity the consortium had set for commercial development, the assay specifically identified *M. tuberculosis*-infected individuals, and provides a valuable proof of concept.

## Background

Although a recent report from World Health Organization (WHO) indicated a decline in the absolute incidence of TB cases, globally tuberculosis (TB) remains one of the most serious causes of infectious disease. It is responsible for about 9 million new cases and more than 1 million deaths (among HIV-negative cases of TB) in 2010 [1]. In addition, *Mycobacterium tuberculosis* is estimated to infect about a third of the world population, and although the vast majority contain the infection without clinical manifestations of the disease [2,3], they are at risk of later reactivation, and can act as reservoir for future disease [4]. The majority of TB cases in developing countries are still diagnosed using smear microscopy and clinical examination, augmented by chest X-ray. The use of X-ray examination is often restricted to advanced pulmonary TB in resource-limited settings, and the sensitivity of smear microscopy is in the range of 22-80%, or even lower in children and extra-pulmonary TB patients [5]. Newly-developed diagnostic tools such as whole blood interferon gamma (IFN-γ) release assays (IGRAs) and real time polymerase chain reaction (PCR)-based GenXpert [4,6-8] are unsuitable for routine clinical use in resource poor settings, where the majority of TB cases occur, as many of these techniques require sophisticated laboratory set-ups, equipment and trained personnel. In particular, the lack of tests that can be used at point of care (POC) is a major impediment to effective TB diagnosis.

In recent years, TB serological tests have drawn attention because of their potential to be developed for POC use. Unlike cell-mediated immune responses, the humoral response to *M. tuberculosis* is often delayed and peaks much later, possibly coinciding with the onset of clinical disease [9]. A specific, sensitive TB serodiagnostic test in a rapid test format could therefore constitute the ideal supplement for current diagnostic approaches in highly TB-endemic countries. Considerable progress has been made in the identification of *M. tuberculosis* antigens with the help of comparative genomics [10-12], and several promising antigens have been identified for use in serodiagnosis [13,14]. The main objective of this study was to screen and evaluate a broad panel of *M. tuberculosis* antigens and identify candidates for construction of an antibody-based immuno-chromatographic lateral flow assay (LFA) in a field-friendly format applicable in point of care screening. The best candidates from the screening panel were then converted to a simple LFA, which could be read without the need for further laboratory equipment, and tested against blood and plasma samples from study cohorts with well-defined clinical status.

## Methods

### Study design and participants

This study was performed on plasma samples collected after written informed consent was obtained from multiple sources, including:

119 HIV-negative TB patients recruited at Danish hospitals,

79 HIV-negative TB patients recruited at Ethiopian hospitals,

99 HIV-negative TB patients recruited at Turkish hospitals,

79 HIV-negative TB patients from Uganda obtained through the WHO/TDR Tuberculosis Specimen bank,

73 close household contacts (HIV-negative) recruited among the families of patients from Ethiopian hospitals,

48 Mantoux-positive, close household contacts (HIV-negative) recruited among the families of patients from Turkish hospitals,

79 non-endemic healthy, HIV-negative controls with no known exposure to TB, recruited from the same communities in Denmark as the TB patients,

58 TB-endemic HIV-negative healthy controls, recruited from the same communities in Ethiopia as the TB patients,

153 TB-endemic HIV-negative healthy controls, recruited from the same communities in Turkey as the TB patients,

8 extra-pulmonary TB patients and 8 symptomatic non-TB community controls, recruited from Turkey and

31 extra-pulmonary TB patients from Ethiopia.

The TB patients were newly diagnosed, bacteriologically confirmed (smear- and/or culture positive, from sputum or needle aspirate) clinical cases with persistent cough for more than 2 weeks, accompanied by signs and symptoms of TB such as productive cough, loss of appetite, weight loss, night sweat or haemoptysis, enlarged lymph nodes or abnormal chest X-ray. Household contacts were defined as individuals who had been living with pulmonary TB (PTB) patients in the same household at least for the past 6 months before recruitment into the study. The majority of these cases were apparently healthy, asymptomatic, and had normal chest X-rays. However, 12 cases had complaints of cough and abnormal chest X-ray (16%), albeit not serious or specific enough for them to be diagnosed as having TB, and were sputum negative. The control groups were apparently healthy volunteers, living in the same community with TB patients. All participants were screened for HIV and were negative. This study received ethical approval from institutional and national ethical review committees of all partners involved: the Statens Serum Institute IRB, København Kommunes Videnskabsetiske Komité in Copenhagen, the Scientific Ethics Committee in Refik Saydam National Hygiene Centre, Ankara and the National Health Science and Technology Council and ethics review committees in Addis Ababa (the AHRI/ALERT Ethics Review Committee and the National Research Ethics Review Committee of Ethiopia).

### Antigen selection and plasma sample preparation

The Statens Serum Institute (SSI) in Denmark produced over 100 TB antigens, identified potential antigens for use in serodiagnosis, and supplied all the antigens evaluated in this study. The bulk of the antigen screening has already been published [15-19] and so will be only briefly described here. From the initial screening process [15-18], we selected 10 antigens, which had demonstrated potential for further analysis. Subsequent rescreening using sera from Denmark, Uganda and Gambia identified 4 candidate antigens (Table 1) that had demonstrated high specificity (95%) and tolerable sensitivity (recognized by over 30% of TB patients). The hope was a combination of antigens would improve the sensitivity of the test as a whole. The antigens selected for the SERO-TB study include a fusion hybrid of the two most characterized proteins, ESAT-6: CFP10 (also called Diaser-3, Rv3875:Rv3874), CFP-8a (Rv3354), ORF-3 (Rv3872) and *MTB*: Acry (Rv2873: Rv2031c). The antigens were expressed in *Escherichia coli* (*E. coli*) vectors and purified as described earlier [15].

**Table 1:** Percent antibody positive responders by ELISA in different population groups to candidate antigens

**Antigen**	**Healthy controls (Denmark, n = 79)**	**TB patients (Denmark, n = 119)**	**TB patients (Uganda, n = 77)**	**Healthy controls (Ethiopia, n = 58)**	**TB patients (Ethiopia n = 79)**
**ESAT-6: CFP10**	**3**	**45**	**91**	**31**	**74**
TB16.3	4	38	56	ND	ND
**MPT83: Acry**	**4**	**72**	**74**	**19**	**64**
CYP15	4	10	30	17	72
CFP21	8	35	50	34	46
Rv1575	18	28	67	50	62
**ORF3**	**5**	**15**	**55**	**16**	**71**
**CFP8A**	**6**	**38**	**74**	**28**	**53**
Rv3118	15	28	72	ND	ND
Rv0222	3	74	89	25	78

A positive result was defined as the negative control (serum without antigen), plus 3 standard deviations as previously described [18]. Those antigens selected for further development in the LFA are written in bold.

Since the antigens were expressed in an *E. coli* vector, all plasma samples were adsorbed against *E. coli* Extract (Promega, San Luis Obispo, CA, USA), to reduce nonspecific responses, according to the manufacturer’s recommendations. Briefly, 20 μl of *E. coli* extract was added to 200 μl of plasma sample, incubated overnight (O/N) at room temperature (RT), with gentle shaking. The day after, the mix was centrifuged at 10,000 rpm for 10 min and the supernatant transferred into new tubes, where it was treated with 0.05% sodium azide to prevent growth of contaminating bacteria. The samples were then kept at 4°C until use. As a control, a subset of plasma samples were tested against the test antigens before and after adsorption (Additional file 1).

In short, antigens were coated in duplicate onto 96-well polystyrene microtiter plates (Nunc, Roskilde, Denmark) at concentrations ranging from 5 to 0. 1 μg/ml in 100 μl of 0.05 M carbonate buffer (pH 9.6) and then incubated overnight at 4°C. After the plates were coated, they were washed three times with phosphate-buffered saline (PBS; pH 7.2)–0.05% Tween 20 (PBS-T), and serum samples diluted 1:100 in 0.2% PBS-T and 1% bovine serum albumin were applied and incubated for 1 hour at room temperature. The plates were then washed with PBS-T, and then incubated for 1 hour with rabbit anti-human immunoglobulin G antibody conjugated with horseradish peroxidase (D0336; Dako, Hillerød, Denmark) diluted 1:1,000 in PBS-T. The plates were washed with PBS-T and enzyme activity was assayed by incubation for 30 min at room temperature with 100 μl of tetramethylbenzidine peroxidase substrate (Bio-Rad, Hercules, Calif.) per well. To stop the reaction, 100 μl of 4 N sulfuric acid was added and the optical density (OD) was measured at 405 nm. In each experimental plate, known concentration of purified human IgG immunoglobulin (rhIgG) (SIGMA) was serially diluted and run as standard (positive control) along with samples and blank wells as negative controls. OD values were then measured, concentrations extrapolated against known concentration of standard (rhIgG) using GraphPad Prism Software (Version 4.0).

### Multi-antigen print immunoassay

Antibody determinations were done by multi-antigen print immunoassay (MAPIA) as described previously [20]. Briefly, the 4 antigens of interest were immobilized on nitrocellulose membrane (Schleicher & Schuell, Keene, NH) at a concentration of 0.05 mg/ml using a semi-automated microsprayer (Linomat IV, Camag Scientific Inc., Wilmington, DE). The membrane was cut perpendicular to the antigen bands into 4 mm wide strips, blocked (1 h) with 1% non-fat skim milk in PBS-T and then incubated for 1 h with serum samples diluted 1:50 in blocking solution. After washing, strips were incubated (1 h) with alkaline phosphatase-conjugated anti-human IgG or anti-human IgM antibody (Sigma Chemical Co., St. Louis, MO) diluted 1:5000 in blocking solution, followed by washing. Bound antibodies were visualized with BCIP/NBT (Kirkegaard & Perry Laboratories, Gaithersburg, MD).

### Lateral flow assay (LFA) production

**Figure 1:**
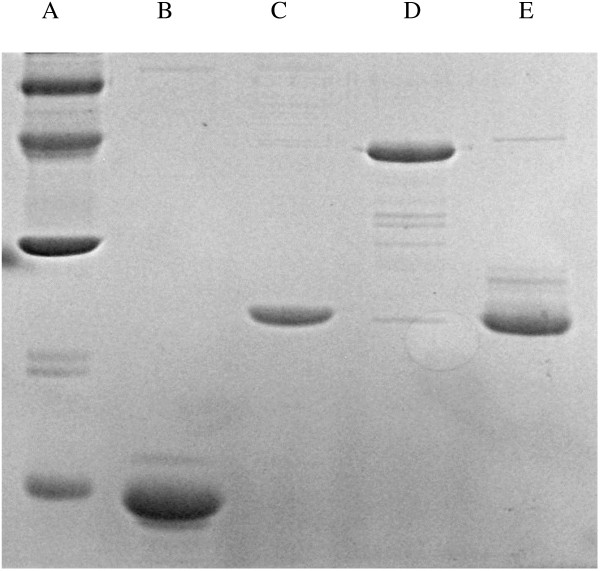
**Recombinant antigens after purification.** SDS-PAGE gel of the recombinant proteins prepared for spraying onto nitrocellulose. A: Molecular weight markers*.* B: ORF3*.* C: ESAT. 6: CFP10*.* D: MPT83: Acry*.* E: CFP8A.

**Figure 2:**
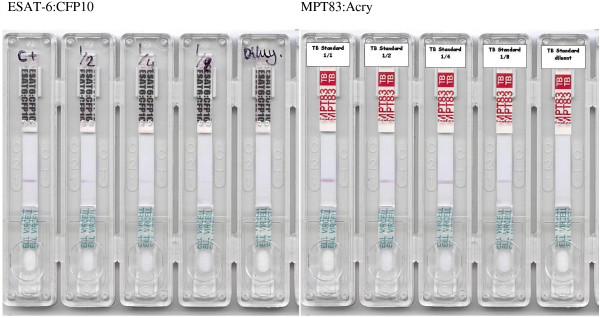
**Serum titration on LFA prototype.** For testing prototypes, a positive control pool of sera from Ugandan TB patients, all with sputum-confirmed tuberculosis and ELISA-confirmed reactivity against one of more of the test antigens was prepared, aliquoted and frozen. A negative control pool was likewise prepared from Danish donors with no known risk factors for TB and no known exposure, all of whom had been confirmed negative for the test antigen by ELISA. A typical result, showing titration of the positive control pool from 1:1 to 1: 8 (left to right), with diluent-only control in the 5th lane of each test to the right.

The clones for recombinant expression of CFP8A, ORF3, CFP10: ESAT-6 and Acry: MPT83 were provided by SSI to Vircell on the pMCT6 plasmid vector, along with the recombinant proteins for comparison and antibodies against the target antigens in order to confirm expression and purification of the correct product. The methods used to generate these clones have been previously described Skjøt et al., 2000, Rosenkrands, 2008. The genes for each of the selected target molecules were transformed into the *E. coli* BL21 strain Rosetta(DE3)pLysS (EMD4Biosciences) to provide expression of the mycobacterial genes which should not be limited by the codon usage of *E. coli*. Since pMCT6 is not optimised as a high yield expression vector, and provided low expression levels in XL1-Blue, the genes were subcloned. For the initial transformations, the expression plasmid pQE60 was used -subsequently, XL1-Blue (Stratagene) transformed with the pREP4 vector (Invitrogen) was used for expression. However, since the yield of some of the recombinant proteins was very low, the heterologous genes were transferred to a different expression vectors in order to improve expression. In the case of ORF3, the expression vector used was pET-15b (Novagen). For CFP8A, an expression vector constructed in Vircell was used, pET-His. Inserted genes in both vectors were under the control of the T7 RNA polymerase promoter and added a hexamer his-tag at the N-terminal of the produced protein. These two new constructions were transformed into the strain of *E. coli* Rosetta(DE3)pLysS, an expression strain which contains a chromosomal copy of the T7 RNAP gene, under the control of the lacUV5 promoter, which can be induced by IPTG. Post-induction, the proteins were collected by centrifugation, followed by lysis of the bacterial pellet and purified under denaturing conditions as previously described [21]. Briefly, denatured proteins were applied to nickel affinity columns (Talon columns; Clontech) and washed with three column volumes of wash buffer (4 M guanidine-HCl, 50 mM sodium phosphate [pH 7.2], 300 mM NaCl). The recombinant proteins were thereafter eluted by adding three column volumes of the wash buffer supplemented with 150 mM imidazole. Flowthrough wash and eluates were collected and selected fractions were pooled after examination on Coomassie blue-stained sodium dodecyl sulfate–polyacrylamide gel electrophoresis (SDS–PAGE) gels (Figure 1). All proteins were then dialyzed against a 20–50 mM phosphate buffer pH 7.5 with 150 mM NaCl to a final concentration of 1 μg/ml, and sprayed onto a nitrocellulose membrane using a Linomat IV microsprayer (Camag Scientific Inc.) at 1 μl/cm^2^. The membrane was dried overnight at 37°C, attached to an adsorbent pad at one end (to wick fluid) and to a conjugate pad overlapped by an absorbent sample pad at the other end. The membrane and absorbent pad unit were immobilized in a plastic case (see Figure 2).

### Application of selected antigens to the lateral-flow format

**Figure 3:**
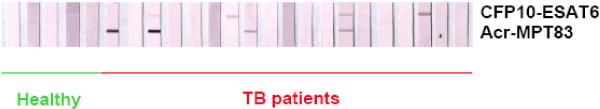
**Antigen screening using multi-antigen print immunoassay.** Staining of antigen printed onto nitrocellulose by sera from 5 negative controls (Danish participants, no known TB exposure of TB risk factors) and 19 patients with confirmed TB, recruited from Danish hospitals. The nitrocellulose strips were incubated with sera diluted 1/50 in 1% non-fat skim milk in PBS-T.

The LFA works on the principle of labelling and capturing antibodies from a blood or serum sample applied as they flow through a membrane and concentrating the label on a line of antigen bound to the membrane so that it becomes visible. If the serum contains antigen-specific antibody, it will react with the immobilized recombinant protein on the testing line, as it migrates through the nitrocellulose membrane, to give rise to a purplish band, the intensity of which will be proportional to the concentration of antibody. For a final production version, a control line of anti-human IgG would have been incorporated at the end of the nitrocellulose to ensure that the sample fluid had crossed the antigen line(s) and as a control on manufacturing quality and that the serum applied was of human origin, but in the test version, this was omitted and fluid transfer simply checked by eye. In addition, multiple antigens would be applied, to increase the sensitivity of the test. Based on initial screening, four antigens with sensitivity of greater than 50% in TB patients from TB-endemic regions were selected to form the basis of the test, which when combined, were predicted to give better than 90% responsiveness among TB patients (Table 1, [15]) while retaining a high degree of specificity. Initial screening using a multi-antigen print immunoassay (MAPIA) ensured that the antigens were also recognised when bound to nitrocellulose (Figure 3). Initial test results indicated that the prototype LFA gave a clearly positive result with each antigen, though not all TB patients tested in the initial MAPIA screen (Danish TB patients, n = 10) responded to all antigens, and no reactivity was seen with negative controls (Danish BCG-vaccinated negative controls, n = 15). However, since the goal of this study was to assess antigen potential for inclusion in a LFA, each antigen was initially tested separately in the LFA. Thus, 100 μl of the serum specimen diluted in PBS was added to the sample chamber and after 15 min, the results were considered positive (if a band was present at the line where the antigen had been applied. In all cases, the most intense staining was seen with sera from TB patients at the lowest dilution (Figure 2). Specificity was assessed the same way using 100 control sera from Jaén (Spain) and El Ejido (Almeria, Spain). The use of negative controls reduced the possibility of positive results in the control group due to unidentified TB exposure. We saw little evidence of non-specific staining by non-TB patients at any serum dilution – only three controls were positive against any of the antigens and the staining seen was weak. A study to determine if there was a prozone effect, using concentrated monoclonal antibodies against the antigen targets showed no such effect (Additional file 2). A study was also conducted to determine the effect of interference due to hemoglobin (which can be a potential problem if lysis of the red blood cells occurs during a blood draw). Different dilutions of hemoglobin (up to 500 mg/dl) were tested, together with the negative and positive control antibody pools. Hemoglobin did not affect the immunological reaction, or its visibility, though at higher concentrations it did produce generalized pinkish staining of the membrane (Additional file 3).

Once the basic parameters were established, the prototype LFAs was assessed for stability and reproducibility in use. Stability was tested at room temperature and at 2-8°C as well as via an accelerated stability study. The stability studies at room temperature (20-25°C) were assessed at 15 days, 1 month, 3 months and then every subsequent three months for 18 months. The performance of the kit at 2-8°C was tested every three months for 18 months. The accelerated stability study was carried out by incubating the LFAs at 37°C and 55°C in a humid atmosphere. Testing was performed at 3, 7, 10, 15, 21, and 30 days at 37°C as well as at 3, 7, 10, and 15 days at 55°C. On each of the indicated days, dilutions of the positive sera pool (from 1:1 to 1:16) were analyzed in duplicate alongside the undiluted negative control pool. For LFAs stored at room temperature, 2-8°C or at 37°C, there was no significant decay of the signal during the testing period, while at 55°C, the intensity of the signal decreased after the seventh day, such that samples at or below 1/4 dilution were clearly weaker in color intensity than the same samples assessed earlier in the study. Finally, reproducibility was tested on three different days, performed with three different sample titrations, and a positive and negative control. The prototype showed excellent reproducibility across different days, samples and operators (Additional file 4). Following these assessments, the LFA test kits were sent to Turkey and Ethiopia for testing under field conditions.

### Data analysis

Raw data was measured as OD values and the concentration of IgG in each sample was extrapolated from the standard curve drawn from human rhIgG standard, using GraphPad Prism Software (Version 4.0), with assumption of a non-linear regression model and Sigmoidal-dose response (variable slope) equation. Comparison between groups was done using one-way ANOVA with the assumption of Kruskal-Wallis test statistics. A *p-value* < 0.05 was taken as indicating statistical significance.

## Results

### Results of the lateral flow test under field conditions

Reports from both sites indicated that the test was well accepted by staff, being simple to use, requiring little extra equipment and producing results after 20–30 minutes. Blood was taken by thumb prick into a 1.5 ml eppendorf tube, allowed to clot, and 50 μl of the sera taken with a micropipette. This was added to the LFA, followed by 50 μl of PBS. The test was then allowed to stand for 15 minutes, at which point, the results were read. The results from Turkey, where the LFA was tested with sera from 99 TB patients, 48 household contacts and 153 community controls are presented in Table 2. The association of positivity with presumed exposure to TB (Table 2) suggests that the LFA retained the high degree of specificity seen in laboratory testing. However, the overall sensitivity was disappointing, with only 46% of the TB patients testing as positive for any one antigen – as compared to 93% who were positive for the Mantoux test at a cut-off of 15 mm. Of the TB household contacts, 38% were positive for at least one antigen (versus 100% positive by Mantoux), while for the community controls 12% were positive for at least one of the antigens in the LFA, versus 5% in the Mantoux test. As can be seen in Table 2, the majority of positive responses were directed against the MPT83: Acry fusion molecule. Although the sample size is too small to draw conclusions, among the 99 TB patients were 8 with extra-pulmonary TB. Of these, only 2 gave a positive test in the LFA – both to the MPT83: Acry fusion molecule.

**Table 2:** Individuals responding to candidate antigens from clinical cohorts in Turkey and Ethiopia

**Results from Turkish clinical cohorts**						
**Cohort**	**Clinical definition**	**Number (percentage) responding to antigen**				
		**ESAT-6**	**CFP8A**	**Acr: MPT83**	**ORF3**	**Any antigen**
TB patients (n = 99)	Pulmonary (n = 91)	17 (18.6%)	9 (9.8%)	40 (43.9%)	5 (5.5%)	45 (45.5%)
	Extra-pulmonary (n = 8)	0 (0%)	0 (0%)	2 (25%)	0 (0%)	2 (25%)
TB contacts (n = 48)	PPD+, known exposure (n = 48)	4 (8.5%)	2 (4.2%)	16 (33.3%)	2 (4.2%)	18 (37.5%)
Community controls (n = 153)	Symptomatic, non-TB (n = 8)	0 (0%)	0 (0%)	0 (0%)	0 (0%)	0 (0%)
	Healthy, no identified exposure (n = 145)	6 (4.2%)	2 (1.4%)	15 (9.1%)	1 (0.7%)	18 (11.8%)
**Results from Ethiopian clinical cohorts**						
**Cohort**	**Clinical definition**	**Number (percentage) responding to antigen**				
		**ESAT-6**	**CFP8A**	**Acr: MPT83**	**ORF3**	**Any antigen**
TB patients (n = 79)	Pulmonary (n = 48)	9 (18.8%)	5 (10.4%)	24 (50.0%)	0 (0%)	26 (54.2%)
	Extra-pulmonary (n = 31)	4 (12.9%)	2 (0.65%)	12 (38.7%)	0 (0%)	12 (38.7%)
TB contacts (n = 73)	Known exposure (n = 73)	4 (8.48%)	2 (4.16%)	19 (26.0%)	0 (0%)	21 (28.8%)
Community controls (n = 60)	Healthy, no identified exposure (n = 60)	6 (4.19%)	2 (1.39%)	12 (20.0%)	0 (0%)	16 (26.7%)

In Ethiopia, the results were very similar. Of the 79 index cases, 48% were positive for one or more antigens, compared to 29% of household contacts and 27% of community controls. The Ethiopian TB patient cohort included 48 pulmonary tuberculosis patients and 31 extra-pulmonary tuberculosis patients with needle-aspirate confirmed tuberculosis lymphadenitis, but although the percentage of responders were lower in the latter group, there were no clear differences between the groups in terms of antigen recognition in the LFA. The observation that a significant number of healthy controls were positive for one or more antigens in the LFA is the only point at which the Ethiopian data diverge from the Turkish data set, and this is consistent with earlier studies in this highly TB-endemic community, which have suggested that over 30% of adult Ethiopian community controls may be latently infected [22]. Mantoux results are not available from Ethiopia as this test is not routinely used in the local clinics, but the results suggest that the test does not differentiate latent and acute *M. tuberculosis* infection. Again, in all cohorts MPT83: Acry was by far the most frequently recognised target, which was unexpected, given the high seropositivity to ESAT-6: CFP10 observed in this population by ELISA [15]. The results (Tables 1 and 2) suggest that the findings seen with the LFA are a result of this particular test formulation’s technical performance, not due to an absence of specific antibody in the participants in the study.

## Discussion

In resource-poor settings, the diagnosis of TB is primarily based on identification of mycobacteria by sputum smear and on clinical evidence. However, bacteriological examination is often time-consuming and relatively insensitive. Better tools are available, but are often impractical due to their cost, technical requirements or the long waiting times required for the diagnostic result. Although the potential for antibody-based diagnostic tools to give a quick answer using a simple blood draw has made them attractive alternatives for TB diagnosis, current test formats have proven ineffective, due to their low sensitivity and specificity [9] and the fact that many of them still require laboratory infrastructure. Concern over the poor accuracy of available commercial serological tests has been raised by the WHO [23,24]. The single most important factor for the failure of the current serological tests is the choice of antigens used, the majority of which are shared with other mycobacteria leading to low specificity in individuals living in the developing countries. Many of the antigens are also relatively weakly recognized, accounting for the poor sensitivity of these tests.

We have addressed these problems systematically, by screening a broad panel of antigens and select those which were best recognized [15-18]. We also identified that antigens that had limited specificity for *M. tuberculosis* infection, by screening with negative controls from both TB-endemic and non-endemic countries. The selected antigens were used to develop an LFA, with the goal of providing a serological test that was inexpensive, simple and suitable for point of care use. Since issues regarding sensitivity and specificity are the major factors that determine the applicability of such test kits [25], particularly in resource limited settings, validation of such tools in different settings is warranted.

Several important observations have been made in the course of this work. Consistent with prior reports [15] although a higher percentage of TB patients had a significant antibody response to the specific antigens chosen for the LFA in the ELISA, there were still a significant number of healthy controls from TB-endemic populations who made positive responses to these antigens (Table 1). Given that prior studies have indicated a high rate of latent infection in this population [22] and the low percentage of responders to these antigens among healthy controls from a non-TB-endemic country, it is likely that this response is indicative of latent TB infection, rather than cross-reactivity. Nonetheless, it poses a problem for using such tests in TB-endemic settings, where the ideal would be to identify active or incipient TB, not just *M. tuberculosis* infection.

Interestingly, the hierarchy of responses observed in ELISA did not predict the outcome in the LFA. When ranked in terms of the percentage of TB patients who made a significant antibody response, TB patients from TB-endemic regions (Uganda and Ethiopia) responded most frequently in the ELISA to the ESAT-6: CFP10 dimer, with a lower percentage responding to the MPT83: Acry dimer. When testing these same antigens in the LFA, this response was completely reversed, with a greatest number of responders being found to the MPT83: Acry dimer. It is not clear if this is a question of avidity, conformational change when the antigens are bound, or some as yet unidentified interference, but it strongly suggests that screening results using ELISA may not always be predictive for other assay formats, including LFA. In addition, we did not recruit sufficient HIV positive individuals to conduct any meaningful analyses in this study, but given the high risk of TB in HIV positive individuals, this is certainly an area that should be further explored.

## Conclusions

The results presented here represent one of the few reports on the testing of an LFA designed to diagnose human TB and provide an important proof of concept. It should be noted that despite the relatively low sensitivity, the test does appear to have a high specificity (over 95%, when assessed against the negative controls from Denmark and Spain, who can be safely assumed to be mostly free of *M. tuberculosis* infection). Since the molecules most frequently recognised in these studies (ESAT-6: CFP10 and MPT83: Acry) gave a sensitivity of over 90% in an LFA developed for primate use [26], these results suggest that with further optimisation, a point of care LFA test could be developed for rapid diagnosis of human TB, perhaps using different antigens [27,28]. It has been suggested that allowing serum antibodies to bind to antigen prior to detecting the antibody-antigen complexes as a second step (as opposed to having antibodies interact with the conjugate prior to antigen binding, as occurs in this assay), can improve the sensitivity of the assay and it may also be possible to improve the signalling conjugate used. Given the ongoing need for a simple, inexpensive point of care test for TB, these options should be further investigated.

## Competing interests

The authors declare that they have no competing interests.

## Authors’ contributions

LW and MA participated in the study design, carried out the immunoassays, analyzed the data and drafted the manuscript, AA conceived the study, participated in its design and assisted in the draft of the manuscript, KB, MZ and MC carried out the immunoassays and assisted in the draft of the manuscript, LKY analyzed the data and assisted in the draft of the manuscript, AC, JRG, JMD, IC, IR and KW participated in the study design, carried out the development of the LFA and prepared the data, PA and TMD conceived the study and participated in its design and coordination and assisted in the draft of the manuscript. All authors read and approved the final manuscript.

## Supplementary Material

Additional file 1 **As a control, a subset of plasma samples were tested against the test antigens before and after adsorption: no significant differences were seen.** As an example, the next tables gather the results corresponding to operator#1. The test strips (here identified as T#1, T#2, etc) were provided unlabelled, so that the operator was not aware of which tests were being read. The notation used reflects scoring against the test strip (marked 2, 1, 0.5 and 0 in order of decreasing intensity), where an arrow is used to indicate slightly greater, or slightly less than, the indicated value. Click here for file

Additional file 2 A study to determine if there was a prozone effect, using a concentrated monoclonal antibodies against the antigen targets showed no such effect (typical response using monoclonal Ab against MPT83 titrated from 1/1-1/16 shown: strip with ORF3 used as negative control). Click here for file

Additional file 3 Hemoglobin did not affect the immunological reaction, or its visibility, though at higher concentrations it did produce generalized faint pinkish staining of the membrane (Typical result at highest concentration shown below). Click here for file

Additional file 4 **Reproducibility was tested by three different operators at three different days each, performed with three different levels (positive sample dilutions), and a positive and negative control respectively.** The prototype showed a good reproducibility between days, samples and operators. As an example, the next tables gather the results corresponding to operator#1. The test strips (here identified as T#1, T#2, etc) were provided unlabelled, so that the operator was not aware of which tests were being read. The notation used reflects scoring against the test strip (marked 2, 1, 0.5 and 0 in order of decreasing intensity), where an arrow is used to indicate slightly greater, or slightly less than, the indicated value. Click here for file
