# Supplementary material for: Development of a proof of concept immunochromatographic lateral flow assay for point of care diagnosis of Mycobacterium tuberculosis
Source: BMC Res Notes. 2013 May 21;6:202. doi: 10.1186/1756-0500-6-202 (PMC3680158; doi:10.1186/1756-0500-6-202)
Supplement: Additional file 1 — As a control, a subset of plasma samples were tested against the test antigens before and after adsorption: no significant differences were seen. As an example, the next tables gather the results corresponding to operator#1. The test strips (here identified as T#1, T#2, etc) were provided unlabelled, so that the operator was not aware of which tests were being read. The notation used reflects scoring against the test strip (marked 2, 1, 0.5 and 0 in order of decreasing intensity), where an arrow is used to indicate slightly greater, or slightly less than, the indicated value. [file 1756-0500-6-202-S1.doc]

As a control, a subset of plasma samples were tested against the test antigens before and after adsorption: no significant differences were seen. As an example, the next tables gather the results corresponding to operator#1. The test strips (here identified as T#1, T#2, etc) were provided unlabelled, so that the operator was not aware of which tests were being read. The notation used reflects scoring against the test strip (marked 2, 1, 0.5 and 0 in order of decreasing intensity), where an arrow is used to indicate slightly greater, or slightly less than, the indicated value.

| **Before (Acryl: MPT83 +)** | | | | | | |
| --- | --- | --- | --- | --- | --- | --- |
|  | **Lot #1** | | **Lot #2** | | **Lot #3** | |
| **Dilution** | **T#1** | **T#2** | **T#1** | **T#2** | **T#1** | **T#2** |
| **1/_1_** | 2 | 2 | 2 | 2 | 2 | 2 |
| **½** | 1↑ | 1↑ | 1 | 1 | 1↑ | 1↑ |
| **¼** | 1↓ | 1↓ | 1↓ | 1↓ | 1 | 1↓ |
| **1/_8_** | 0.5↑ | 0.5↑ | 0.5↑ | 0.5 | 0.5↑ | 0.5↑ |
| **After (Acryl: MPT83 +)** | | | | | | |
|  | **Lot #1** | | **Lot #2** | | **Lot #3** | |
| **Dilution** | **T#1** | **T#2** | **T#1** | **T#2** | **T#1** | **T#2** |
| **1/_1_** | 2↓ | 2↓ | 2 | 2 | 2↓ | 2↓ |
| **½** | 1↑ | 1↑ | 1 | 1 | 2↓ | 1↑ |
| **¼** | 1 | 1↓ | 1↓ | 1↓ | 1↑ | 1 |
| **1/_8_** | 0.5↑ | 0.5↑ | 0.5 | 0.5 | 1 | 0.5↓ |
