# Supplementary material for: Development of a proof of concept immunochromatographic lateral flow assay for point of care diagnosis of Mycobacterium tuberculosis
Source: BMC Res Notes. 2013 May 21;6:202. doi: 10.1186/1756-0500-6-202 (PMC3680158; doi:10.1186/1756-0500-6-202)

A study to determine if there was a prozone effect, using a concentrated monoclonal antibodies against the antigen targets showed no such effect (typical response using monoclonal Ab against MPT83 titrated from 1/1-1/16 shown: strip with ORF3 used as negative control).


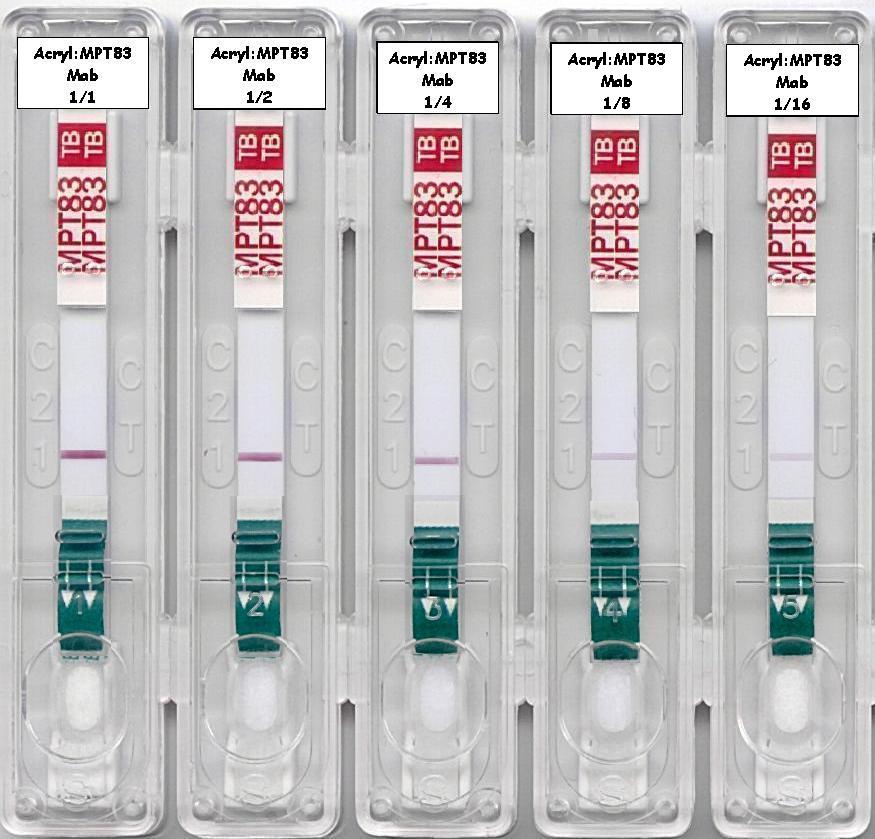

Supplement: Additional file 2 — A study to determine if there was a prozone effect, using a concentrated monoclonal antibodies against the antigen targets showed no such effect (typical response using monoclonal Ab against MPT83 titrated from 1/1-1/16 shown: strip with ORF3 used as negative control). [file 1756-0500-6-202-S2.doc]
