# Supplementary material for: Development of a proof of concept immunochromatographic lateral flow assay for point of care diagnosis of Mycobacterium tuberculosis
Source: BMC Res Notes. 2013 May 21;6:202. doi: 10.1186/1756-0500-6-202 (PMC3680158; doi:10.1186/1756-0500-6-202)

Hemoglobin did not affect the immunological reaction, or its visibility, though at higher concentrations it did produce generalized faint pinkish staining of the membrane (Typical result at highest concentration shown below).


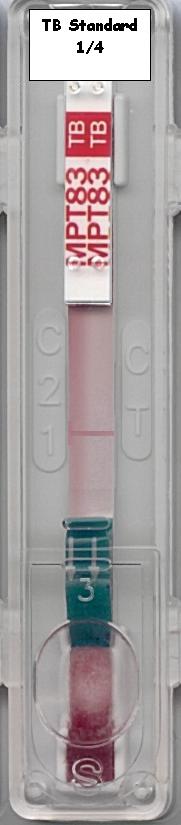

Supplement: Additional file 3 — Hemoglobin did not affect the immunological reaction, or its visibility, though at higher concentrations it did produce generalized faint pinkish staining of the membrane (Typical result at highest concentration shown below). [file 1756-0500-6-202-S3.doc]
