# Supplementary material for: Development of a proof of concept immunochromatographic lateral flow assay for point of care diagnosis of Mycobacterium tuberculosis
Source: BMC Res Notes. 2013 May 21;6:202. doi: 10.1186/1756-0500-6-202 (PMC3680158; doi:10.1186/1756-0500-6-202)
Supplement: Additional file 4 — Reproducibility was tested by three different operators at three different days each, performed with three different levels (positive sample dilutions), and a positive and negative control respectively. The prototype showed a good reproducibility between days, samples and operators. As an example, the next tables gather the results corresponding to operator#1. The test strips (here identified as T#1, T#2, etc) were provided unlabelled, so that the operator was not aware of which tests were being read. The notation used reflects scoring against the test strip (marked 2, 1, 0.5 and 0 in order of decreasing intensity), where an arrow is used to indicate slightly greater, or slightly less than, the indicated value. [file 1756-0500-6-202-S4.doc]

Reproducibility was tested by three different operators at three different days each, performed with three different levels (positive sample dilutions), and a positive and negative control respectively. The prototype showed a good reproducibility between days, samples and operators. As an example, the next tables gather the results corresponding to operator#1. The test strips (here identified as T#1, T#2, etc) were provided unlabelled, so that the operator was not aware of which tests were being read. The notation used reflects scoring against the test strip (marked 2, 1, 0.5 and 0 in order of decreasing intensity), where an arrow is used to indicate slightly greater, or slightly less than, the indicated value.

|  | **TB standard (Acryl: MPT83 +)** | | | | | | | | |
| --- | --- | --- | --- | --- | --- | --- | --- | --- | --- |
|  | **Day 1** | | | **Day 2** | | | **Day 3** | | |
| **Dilution** | **T#1** | **T#2** | **T#3** | **T#2** | **T#1** | **T#3** | **T#2** | **T#1** | **T#3** |
| **½** | 1 | 1↑ | 1 | 1 | 1↑ | 1↑ | 1↑ | 1↑ | 1↑ |
| **¼** | 1↓ | 1↓ | 1↓ | 1↓ | 1 | 1↓ | 1↓ | 1 | 1↓ |
| **1/_8_** | 0.5 | 0.5↑ | 0.5↑ | 0.5 | 0.5↑ | 0.5↑ | 0.5↓ | 0.5↑ | 0.5↓ |

| **Positive control. TB standard (Acryl: MPT83 +)** | | | | | | | | |
| --- | --- | --- | --- | --- | --- | --- | --- | --- |
| **Day 1** | | | **Day 2** | | | **Day 3** | | |
| **T#1** | **T#2** | **T#3** | **T#2** | **T#1** | **T#3** | **T#2** | **T#1** | **T#3** |
| 2 | 2↓ | 2 | 2 | 2↓ | 2↓ | 2 | 2↓ | 2 |

| **Negative control TB** | | | | | | | | |
| --- | --- | --- | --- | --- | --- | --- | --- | --- |
| **Day 1** | | | **Day 2** | | | **Day 3** | | |
| **T#1** | **T#2** | **T#3** | **T#2** | **T#1** | **T#3** | **T#2** | **T#1** | **T#3** |
| 0 | 0 | 0 | 0 | 0 | 0 | 0 | 0 | 0 |
